# The Effect of Kefir Consumption on Blood Pressure and C‐Reactive Protein: A Systematic Review and Meta‐Analysis of Randomised Controlled Trials

**DOI:** 10.1002/edm2.70124

**Published:** 2025-10-26

**Authors:** Elaheh Rashidbeygi, Mahnoush Mehrzad Samarin, Fatemeh Sheikhhossein, Amir Hossein Khalilkhaneh, Masoomeh Gholizadeh, Negin Lohrasbi, Amin Abbasi, Hadi Bazyar, Gholamreza Askari, Mohammad Reza Amini

**Affiliations:** ^1^ Department of Clinical Nutrition and Dietetics, Faculty of Nutrition Sciences and Food Technology, National Nutrition & Food Technology Research Institute Shahid Beheshti University of Medical Sciences Tehran Iran; ^2^ Master of Sport Physiology, Education and Sport Science Department of Physical Education and Sport Sciences, Science and Research Branch Islamic Azad University Tehran Iran; ^3^ Department of Clinical Nutrition, School of Nutritional Sciences and Dietetics Tehran University of Medical Sciences (TUMS) Tehran Iran; ^4^ Department of Human Nutrition, School of Medicine Urmia University of Medical Sciences Urmia Iran; ^5^ Student Research Committee, Department of Clinical Nutrition & Dietetics, National Nutrition & Food Technology Research Institute Shahid Beheshti University of Medical Sciences Tehran Iran; ^6^ Department of Food Science and Technology, Faculty of Nutrition and Food Sciences Tabriz University of Medical Sciences Tabriz Iran; ^7^ Department of Public Health Sirjan School of Medical Sciences Sirjan Iran; ^8^ Student Research Committee Sirjan School of Medical Sciences Sirjan Iran; ^9^ Nutrition and Food Security Research Center and Department of Community Nutrition, School of Nutrition and Food Science Isfahan University of Medical Sciences Isfahan Iran; ^10^ Nutrition and Food Security Research Center, School of Nutrition and Food Science Isfahan University of Medical Sciences Isfahan Iran

**Keywords:** blood pressure, kefir, meta‐analysis, randomised clinical trial

## Abstract

**Background:**

Kefir, a traditional fermented milk beverage, has been increasingly promoted for its various health benefits. This systematic review and meta‐analysis of randomised controlled trials (RCTs) aimed to assess the effects of kefir on blood pressure and C‐reactive protein (CRP) levels.

**Methods:**

A literature search was conducted using databases such as ISI Web of Science, Scopus and PubMed for articles published until January 2025, with no restrictions. A random‐effects meta‐analysis was conducted for all key outcome measures. The inverse‐variance weighted mean difference (WMD) was calculated with a 95% confidence interval (CI).

**Results:**

A total of seven RCTs, comprising 385 subjects, were included in the meta‐analysis. We involved RCTs conducted on adult participants (over 18 years old). These studies generally administered kefir for at least 2 weeks and compared the outcomes to those in the control group. The findings showed that kefir consumption had no significant impact on systolic blood pressure (SBP) (WMD: −1.76 mmHg; 95% CI: −5.21, 1.69; *p* = 0.317), diastolic blood pressure (DBP) (WMD: −1.19 mmHg; 95% CI: −3.40, 1.03; *p* = 0.295) or CRP levels (WMD: −0.17 mg/L; 95% CI: −0.84, 0.49; *p* = 0.609) compared to those who did not consume kefir. However, subgroup analysis indicated that CRP levels significantly decreased with longer durations of kefir consumption (≥ 8 weeks).

**Conclusion:**

Kefir consumption in adults did not result in significant reductions in systolic or diastolic blood pressure or CRP levels. Nonetheless, there is some evidence that long‐term kefir consumption may improve CRP levels over time.

## Graphical abstract

We examined the effects of kefir consumption on blood pressure and C‐reactive protein. Kefir consumption had no significant impact on SBP, DBP and CRP. CRP levels significantly decreased with longer durations of kefir consumption (≥ 8 weeks).

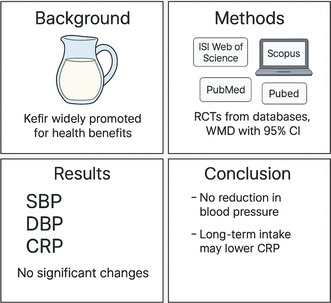

## 1 Introduction

Over the past two decades, the use of evidence‐based complementary and alternative medicine has dramatically expanded in the healthcare sector, especially in societies with traditional beliefs, to reduce the risk of stroke, ischemic heart disease, cardiovascular mortality, type 2 diabetes mellitus and the development of metabolic syndrome [1, 2, 3]. Kefir is a drink with an acidic taste and creamy consistency that can be prepared from any type of milk, such as goat, buffalo, sheep, camel, cow, coconut, rice or soy via bacterial fermentation (inoculating milk with kefir grains) [3, 4]. The literature review demonstrates that there is a growing public interest in this fermented beverage with low alcohol content as a functional food, owing to several important nutraceutical benefits and being safe and inexpensive [1, 5].

The best‐known member of the pentraxin family is C‐reactive protein (CRP) [6], which is produced by the liver and by adipocytes in response to stress [7]. Elevation in CRP levels has been indicated as a predictor of future cardiovascular events [8]. As an inflammatory mediator, it can stimulate the renin‐angiotensin‐aldosterone cascade and enhance the pro‐atherogenic effects of angiotensin, causing alterations in the functional and structural properties of the vascular wall, enhancement of thickening and stiffening of the vessel wall, endothelial dysfunction and interference with the blood pressure regulation systems [6, 9, 10]. Vascular inflammation plays a primary role in the initiation and evolution of hypertension [11].

Despite widespread knowledge about how to diagnose and define hypertension, and the widespread availability of effective and low‐cost antihypertensive drugs, the percentage of subjects presenting with hypertension increases markedly with age [10, 12]. In 2019, this highly preventable and treatable condition accounted for about one‐fifth of all deaths across all regions of the world [13]. Recently, the hypertension guidelines recommend the adoption of dietary modifications, including salt intake reduction and a healthy diet with higher probiotic content in all subjects with suboptimal blood pressure levels [14, 15, 16]. Kefir grains contain species of yeasts, lactic acid bacteria, acetic acid and mycelial fungi [17]. Most of the purported health benefits are closely related to the microbial populations that are present in kefir, the interplay between its microbial composition, and microbial peptides produced during fermentation, including angiotensin‐converting enzyme (ACE) inhibitory peptides [5, 18]. These peptides inhibit the production of aldosterone and influence the decrease in blood pressure [5, 19]. Promising experimental studies suggest that a high intake of kefir may result in greater reductions in plasma CRP levels and have a positive impact on cardiovascular conditions [20].

Various clinical randomised controlled trials (RCTs) have assessed the benefits of kefir consumption on blood pressure and CRP in different populations, but study results yield inconsistent conclusions [14, 21, 22, 23, 24]. This inconsistency could be attributed to the different sample sizes in these studies, study duration, variable doses and characteristics and comorbidities of the studied population. Hence, a meta‐analysis of clinical trials would be appropriate to reach a conclusive result on the health benefits of the dietary intervention of kefir on blood pressure and CRP. Therefore, the purpose of this meta‐analysis is to provide a comprehensive summary of the effect of kefir consumption on blood pressure and CRP. Furthermore, we performed subgroup analyses to elucidate the essential association between intervention duration and CRP.

## 2 Methods

### 2.1 Systematic Search and Study Selection

This review followed the guidelines for systematic reviews and meta‐analyses (PRISMA) statements [25]. This study's registration number in PROSPERO is CRD420251158732. A systematic literature search was conducted in ISI Web of Science, Scopus and PubMed search engine up to January 13, 2025, with no restrictions on dates, specific language or any other filters. Also, a manual search was conducted by reviewing the first ten pages of Google Scholar and examining the references of relevant review studies. A standard protocol for this search was developed, and the search strategy was the following queries: (kefiran OR kefir) AND (‘Blood Pressure’ OR SBP OR DBP OR CRP) AND (intervention OR randomised OR trials) (Table S1). A further literature search, which screened kefir‐related terms up to January 13, 2025, was conducted using Google Scholar. In addition, we checked reference lists of included studies. Systematic screening was conducted by two authors, (ER and AKH) separately and independently. Data were rechecked by another researcher (MRA).

### 2.2 Experimental Methods

Based on PICOS framework: P (18 years or older), I (kefir), C (control group that received a placebo includes unfermented milk, milk supplementation, curd, non‐kefir products, diet or no treatment), O (systolic blood pressure (SBP), diastolic blood pressure (DBP) and CRP) and S (parallel clinical trials), the title/abstract and full text of eligible studies were examined by two authors independently (ER and AKH). Studies were selected for inclusion if they (1) reported the effects of dietary kefir supplementation on related outcomes with a control group, (2) applied an RCT, (3) were conducted on an adult population (age > 18 years), (4) and administered kefir for at least 2 weeks. Studies were excluded if they were uncontrolled studies, dietary intake of dairy products was not examined during the intervention, and kefir in combination with other supplements, as well as animal and in vitro studies, editorials, reviews and research with inadequate data on associated outcomes.

### 2.3 Data Extraction

The following data were extracted: first author, publication year, country, study design, gender, target population, sample size (control and intervention groups), mean age and body mass index (BMI) of participants in control and intervention groups, duration of intervention, dose, type of kefir and placebo and outcomes. We converted to the same unit and effect size (means ± standard deviations (SDs)) if studies reported different units or any other effect sizes for SBP, DBP and CRP. Data extraction and study selection were carried out separately by two investigators (ER and AKH). Disagreements were settled through conversation between the researchers; if they could not reach an agreement, a third reviewer was consulted (MRA).

### 2.4 Quality Assessment

To evaluate the risk of bias in the included studies, we used Cochrane Collaboration's tool for systematic reviews of interventions [26]. Each item was given a low risk of bias, high risk of bias or unclear risk of bias. The total risk of bias for a trial was determined as good (low risk for more than two items), fair (low risk for two items) and weak (low risk for less than two items) [27]. Two different researchers (ER and AKH) evaluated the quality of the included papers (Table 1).

**TABLE 1:** Risk of bias for randomised controlled trials, assessed according to the Revised Cochrane risk‐of‐bias tool for randomised trials.

Publications	Random sequence generation	Allocation concealment	Selective reporting	Blinding (participants and personnel)	Blinding (outcome assessment)	Incomplete outcome data	Other source of bias
1. Bellikci Koyu (2019) [14]	L	L	L	H	H	L	H
2. Bellikci Koyu (2022) [21]	L	L	L	L	U	L	L
3. Ghizi (2021) [22]	L	L	L	L	U	L	L
4. Gooruee (2024) [23]	L	L	L	L	U	L	L
5. Praznikar (2020) [28]	L	U	L	H	H	L	H
6. Yilmaz (2019) [29]	L	U	L	H	H	L	L
7. Mohammadi (2025) [24]	L	L	L	H	H	L	L

Abbreviations: H, High risk of bias; L, Low risk of bias; U, Unknown.

### 2.5 Statistical Analyses

A random‐effects meta‐analysis was performed to evaluate all primary outcome measures. Weighted mean differences (WMDs) and 95% confidence intervals (CIs) were calculated to assess the effects of kefir on SBP, DBP and CRP [30]. The mean change and SD for each outcome in the kefir and control groups were used to compute pooled effect sizes, following the methodology outlined by Higgins et al. [26]. Standard errors (SEs) were converted to SDs using the formula SD = SE × √*n*, where *n* represents the sample size, as described in [31]. We performed pre‐specified subgroup analyses based on study duration and participants' age to investigate sources of heterogeneity. Begg's [32] and Egger's [33] tests were used to examine publication bias. We utilized sensitivity analysis to assess the impact of each RCT on the final results [34]. Furthermore, we applied Cochran's Q test and I‐squared (*I* ^2^) statistic to find out heterogeneity between the studies [34]. We considered it significant heterogeneity between studies if *p*‐values < 0.05 and *I* ^2^ > 50%. In general, *p*‐value < 0.05 is considered statistically significant. We used STATA software, version 14.0 (StataCorp, College Station, TX, USA) for conducting all tests.

## 3 Results

### 3.1 Literature Search

**FIGURE 1:**
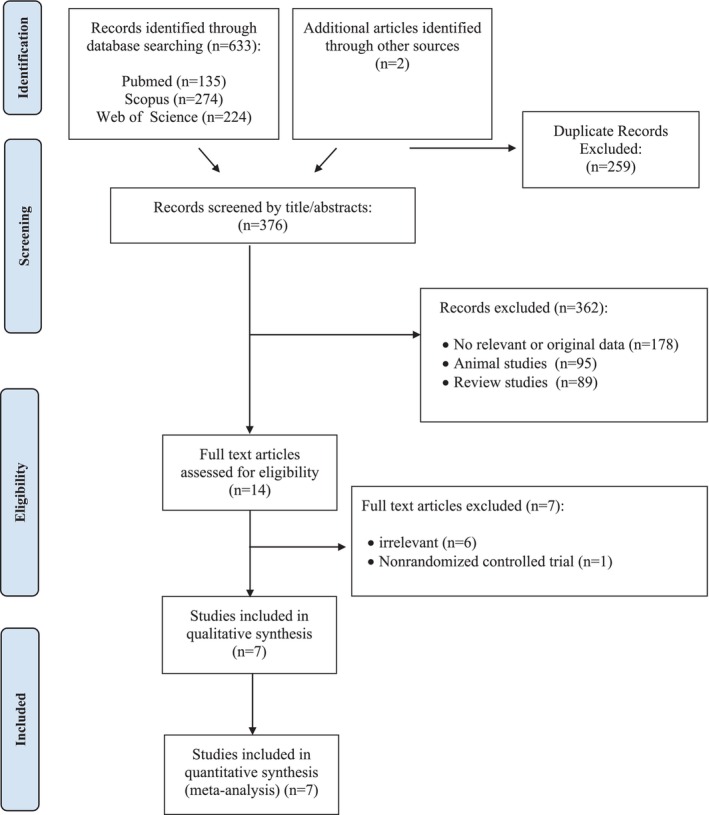
Flow chart of the number of studies identified and selected into the meta‐analysis.

At first, we searched manually and identified 633 records from all the electronic databases. Also, 2 articles were identified through other sources. Then, 259 duplication records and 362 irrelevant studies were excluded through screening based on the title or abstract. Among the 14 remaining publications, 7 articles were excluded after reading the full text since they did not have a relevant outcome (*n* = 6), and in 1 study there was a nonrandomized controlled trial. Overall, 7 studies [14, 21, 22, 23, 24, 28, 29] met the eligibility criteria for the final analysis. The study of Yilmaz et al. (2019) [29] had two effect sizes because the intervention groups were divided into two supplementation groups. The study's identification procedure flow diagram is shown in (Figure 1).

### 3.2 Study Characteristics

Included trials were published between 2019 and 2025. A total of 385 individuals with a mean age of 38–52 years are included in the present Systematic Review and Meta‐analysis. The Control group received a placebo that includes unfermented milk, milk supplementation, curd, non‐kefir products, diet or no treatment. All studies applied a parallel design, and both genders were enrolled in them. The dosage of kefir ranged from 90 – 500 mL per day, and the intervention duration varied between 2 and 12 weeks. Studies were carried out on healthy adults and subjects with metabolic syndrome, ulcerative colitis, Crohn's disease, nonalcoholic fatty liver disease and COVID‐19 patients. Also, these trials were conducted in Turkey [14, 21, 29], Brazil [22], Slovenia [28] and Iran [23, 24]. Table 2 provides an overview of the general features of these RCTs.

**TABLE 2:** Demographic characteristics of the included studies.

First author (year)	Location	Study design	Health status	Sex	Sample size	Duration (week)	Mean age (year)	Baseline BMI (kg/m^2^)	Intervention group	Comparator group	Outcome
1. Bellikci Koyu (2019) [14]	Turkey	RCT, parallel	Metabolic syndrome	Both	22	12	52.5	31.52	180 mL/day kefir drink	Unfermented milk	SBP/DBP/CRP
2. Bellikci Koyu (2022) [21]	Turkey	RCT, parallel	Metabolic syndrome	Both	62	12	49.8	32.6	180 mL/day kefir drink	Unfermented milk	SBP/DBP/CRP
3. Ghizi (2021) [22]	Brazil	RCT, parallel	Metabolic syndrome	Both	48	12	43	31.7	1.6 mL/kg for men or 1.9 mL/kg for women kefir drink	Curd	SBP/DBP/CRP
4. Gooruee (2024) [23]	Iran	RCT, parallel	COVID‐19 patients	Both	100	2	47	25.11	500 mL/day kefir drink	Non‐kefirper	CRP
5. Praznikar (2020) [28]	Slovenia	RCT, crossover	Healthy	Both	28	3	45.8	29.1	300 mL/day kefir drink	Milk supplementation	CRP
6. Yilmaz (a) (2019) [29]	Turkey	RCT, parallel	Ulcerative Colitis	Both	25	4	38.2	NA	400 mL/day kefir drink	No treatment	CRP
7. Yilmaz (b) (2019) [29]	Turkey	RCT, parallel	Crohn Disease	Both	20	4	37.5	NA	400 mL/day kefir drink	No treatment	CRP
8. Mohammadi (2025) [24]	Iran	RCT, parallel	Nonalcoholic fatty liver disease	Both	80	8	42.8	29.7	500 mL/day kefir drink + Diet	Diet	SBP/DBP/CRP

Abbreviations: BMI, Body mass index; CRP, C‐reactive protein; DBP, Diastolic blood pressure; NA, Not available; RCT, Randomised controlled trial; SBP, Systolic blood pressure.

### 3.3 Risk of Bias Assessment

The Cochrane Collaboration's tool indicated that the overall quality score was good and that further studies were of moderate quality. Each trial provided enough information regarding random sequence generation items, selective reporting, and incomplete outcome data. Most studies had a high risk of bias regarding blinding participants, blinding outcomes, and other sources of bias. Also, most studies had a low risk of bias regarding allocation concealment, except Praznikar et al. (2020) [28] and Yilmaz et al. (2019) [29]. Table 1 illustrates the risk of bias in each study.

### 3.4 Effect of Kefir on SBP

**FIGURE 2:**
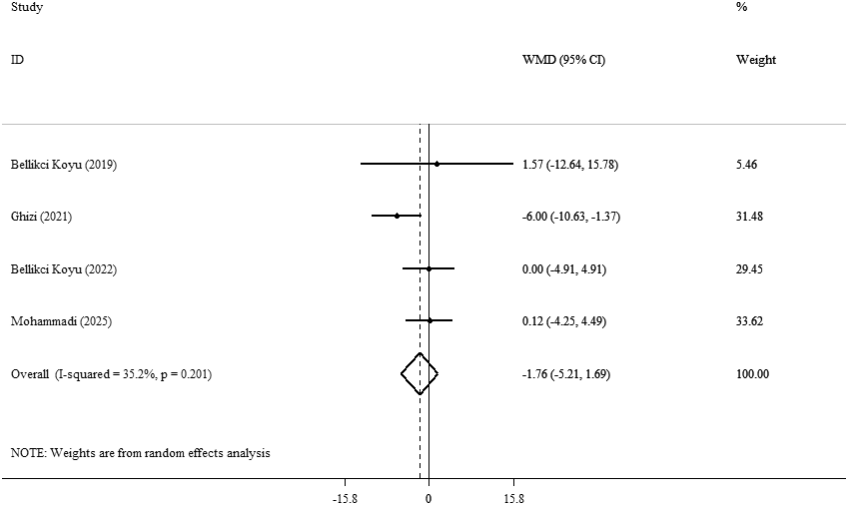
Forest plot detailing weighted mean difference and 95% confidence intervals (CIs) for the effect of kefir consumption on SBP.

Four clinical trials [14, 21, 22, 24] and effect size pooled findings showed that the kefir had no significant impact on SBP (WMDs: −1.76 mmHg; 95% CI: −5.21, 1.69; *p* = 0.317) (Figure 2). Also, low heterogeneity was observed between the trials (*I* ^*2*^ = 35.2%, *p* = 0.201). During the sensitivity analysis, each trial was gradually removed from the pooled analysis to assess the impact of each study on the pooled effect size. The results showed that deleting each trial had no meaningful effect on the WMD. This meant that the meta‐analysis results remained consistent and were unaffected by any of the four trials. To assess publication bias, we used Begg's (*p* = 0.734) and Egger's test (*p* = 0.789). Table 3 presents the results of a meta‐analysis of the effects of kefir supplementation on SBP.

**TABLE 3:** Subgroup analysis of included randomised controlled trials in meta‐analysis of the effect of kefir consumption on blood pressure and C‐reactive protein.

Group	No. of effect size	WMD (95% CI)	*p*	*I* ^2^ (%)	*p*‐heterogeneity	*p* for between subgroup heterogeneity
*SBP*						
Age						
≤ 45	2	−2.76 (−5.94, 0.42)	0.09	71.8	0.06	0.30
> 45	2	0.17 (−4.47, 4.81)	0.94	0.0	0.84	
*DBP*						
Age						
≤ 45	2	−2.22 (−5.62, 1.18)	0.20	0.0	0.80	0.43
> 45	2	−0.42 (−3.35, 2.51)	0.78	0.0	0.72	
*CRP*						
Duration (week)						
≤ 8	5	−0.05 (−0.22, 0.13)	0.58	81.2	< 0.001	0.09
> 8	3	−0.47 (−0.93, −0.02)	0.04	61.3	0.08	
Age						
≤ 45	4	−0.09 (−0.26, 0.08)	0.32	0.0	0.81	0.49
> 45	4	−0.29 (−0.85, 0.27)	0.31	89.3	< 0.001	

Abbreviations: CRP, C‐reactive protein; DBP, Diastolic blood pressure; SBP, Systolic blood pressure; WMD, Weight mean difference.

### 3.5 Effect of Kefir on DBP

**FIGURE 3:**
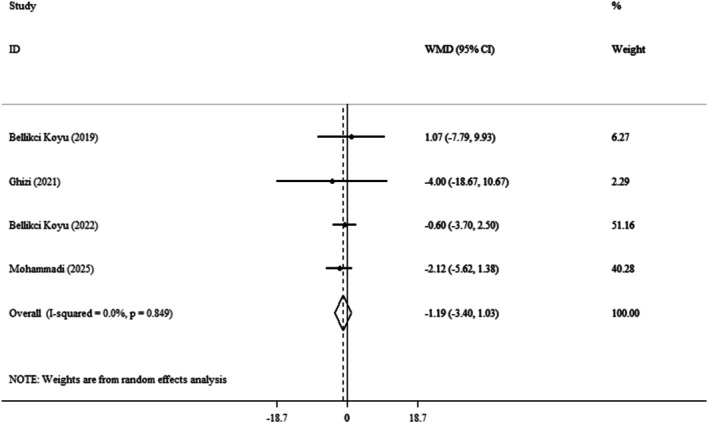
Forest plot detailing weighted mean difference and 95% confidence intervals (CIs) for the effect of kefir consumption on DBP.

Four clinical trials [14, 21, 22, 24] and effect size pooled findings showed that the kefir had no significant impact on DBP (WMDs: −1.19 mmHg; 95% CI: −3.40, 1.03; *p* = 0.295) (Figure 3). Also, heterogeneity was not observed between the trials (*I* ^**2**^ = 0.0%, *p* = 0.849). During the sensitivity analysis, each trial was gradually removed from the pooled analysis to assess the impact of each study on the pooled effect size. The results showed that deleting each trial had no meaningful effect on the WMD. This meant that the meta‐analysis results remained consistent and were unaffected by any of the four trials. To assess publication bias we used Begg's (*p* = 0.734) and Egger's test (*p* = 0.964). Table 3 presents the results of a meta‐analysis of the effects of kefir supplementation on DBP.

### 3.6 Effect of Kefir on CRP

**FIGURE 4:**
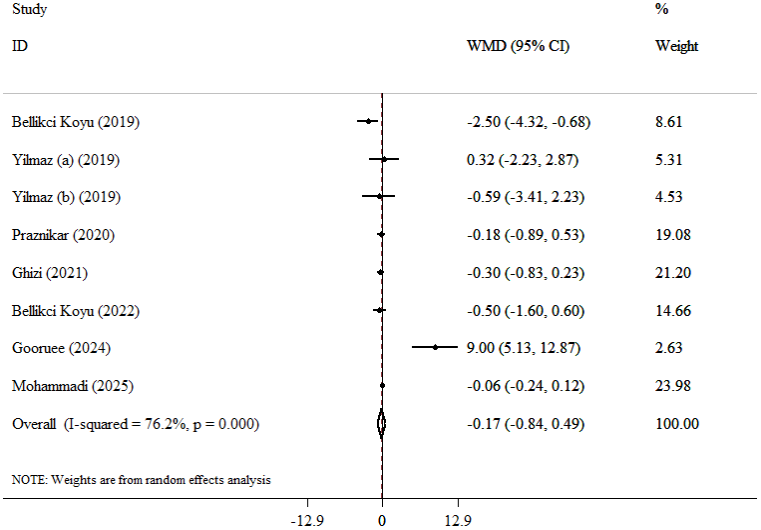
Forest plot detailing weighted mean difference and 95% confidence intervals (CIs) for the effect of kefir consumption on CRP.

Pooled data from seven clinical trials [14, 21, 22, 23, 24, 28, 29] with eight effect sizes demonstrated that the kefir supplementation hadn't a significant impact on CRP (WMDs: −0.17 mg/L; 95% CI: −0.84, 0.49; *p* = 0.609) (Figure 4). Also, high heterogeneity was observed between the trials (*I* ^***2***^ = 76.2%, *p* = 0.0). For this reason, a subgroup analysis was conducted, and the results showed that the intake of kefir for more than 8 weeks (> 8 weeks) can significantly decrease CRP levels (−0.47 mg/L; 95% CI: −0.93, −0.02; *p* = 0.04).

During the sensitivity analysis, each trial was gradually removed from the pooled analysis to assess the impact of each study on the pooled effect size. The results showed that deleting each trial had no meaningful effect on the WMD. This meant that the meta‐analysis results remained consistent and were unaffected by any of the four trials. To assess publication bias we used Begg's (*p* = 0.711) and Egger's test (*p* = 0.867). Table 3 presents the results of a meta‐analysis of the effects of kefir on CRP.

## 4 Discussion

This meta‐analysis aimed to investigate the effects of kefir beverage consumption on blood pressure and CRP. Notably, it is the first systematic review and meta‐analysis to assess these effects in adults. The analysis included seven studies, comprising a total of eight arms and 385 participants. The results of the meta‐analysis indicate that kefir beverage consumption does not have significant effects on blood pressure and CRP when compared to individuals who did not consume kefir. However, subgroup analysis revealed that CRP levels decreased significantly with longer durations of kefir consumption (≥ 8 weeks). The significant reduction in CRP observed with longer durations (≥ 8 weeks) suggests a possible dose–response relationship, where extended kefir intake enhances its anti‐inflammatory effects. This could be due to direct effects of kefir's bioactive compounds—such as peptides, organic acids or bacteriocins—that may accumulate or exert more pronounced effects over time. Alternatively, it might result from gradual improvements in gut microbiota composition and diversity, which can modulate systemic inflammation indirectly. The sustained presence of probiotics and fermentation‐derived metabolites could promote a healthier gut environment, reduce intestinal permeability and thereby lower systemic inflammatory markers like CRP. Highlighting this potential temporal relationship emphasizes that longer‐term kefir consumption might be necessary to observe notable anti‐inflammatory benefits, and it warrants further investigation into the mechanisms and optimal durations for such effects. Although the overall results did not reach statistical significance, the confidence intervals for blood pressure—such as the SBP estimate (−5.21 to 1.69 mmHg)—approach thresholds that could be considered clinically meaningful. Notably, the upper bound of the CI is close to a 5 mmHg reduction, a level often associated with meaningful cardiovascular risk reduction. This suggests that, while definitive conclusions cannot be drawn, there remains a possibility that kefir may exert modest beneficial effects on blood pressure that warrant further investigation. The lack of significant changes in blood pressure and CRP observed in our meta‐analysis may be attributed to several factors including insufficient duration, inadequate amounts of live probiotics per gram of kefir consumed in the studies, and differences in how individuals respond to the beneficial bacteria in kefir could also significantly influence outcomes.

A recent meta‐analysis revealed that consuming fermented dairy products is linked to notable decreases in specific inflammatory biomarkers [3]. Kefir is categorised as a type of fermented milk according to food standards, created using a starter culture derived from kefir grains. These grains consist of *Lactobacillus kefiri* and various species from the *Leuconostoc*, *Lactococcus* and *Acetobacter* genera, which maintain a strong and specific symbiotic relationship. Additionally, kefir grains contain both lactose‐fermenting yeasts, such as *Kluyveromyces marxianus*, and non‐lactose‐fermenting yeasts, including *Saccharomyces unisporus*, *Saccharomyces cerevisiae* and *Saccharomyces exiguus* . Importantly, kefir is abundant in a live culture of bacteria, yeasts and fungi, as well as kefiran (a water‐soluble polysaccharide), unique peptides, organic acids and bacteriocins. These elements are associated with various health benefits, such as improvements in lipid profiles, antimicrobial properties and anti‐inflammatory effects [35]. There were no significant changes in systolic and diastolic blood pressure in the present study. These results are consistent with those from studies with probiotic yogurt interventions [36, 37]. The findings of recent studies align with our research, indicating that kefir consumption does not have positive effects on SBP and DBP [14, 24]. For instance, a study examining the impact of kefir on hypertensive individuals found no significant changes in DBP, although there was a noted decrease in SBP. However, this change was not statistically significant when compared to the control group [24]. Additionally, another study focused on the effects of kefir on metabolic syndrome parameters reported that while some health benefits were observed, there were no significant differences in blood pressure measurements between the kefir and control groups [14]. Contrary to the findings of the present study, several investigations have shown that kefir intervention in rats can lead to a reduction in blood pressure [38, 39]. The antihypertensive effects of kefir may be attributed to improvements in gut health and reductions in neuroinflammation. The treatment was associated with changes in the intestinal wall structure and a decrease in inflammatory markers in the brain regions that regulate cardiovascular function [40]. Another study highlighted that chronic administration of kefir improved endothelial function in SHRs, which is crucial for maintaining vascular health. This improvement was linked to a better balance between reactive oxygen species and nitric oxide, contributing to lower blood pressure [41]. The beneficial effects of kefir are attributed to the release of various bioactive compounds during fermentation, including peptides, lipids and other substances that can enhance health [19, 42]. Kefir contains bioactive peptides that are released during the fermentation process. These peptides have been shown to exhibit various health benefits, including antihypertensive, antioxidant, and anti‐inflammatory properties. They may also play a role in modulating the immune system and improving gut health [43]. In a recent study, Amorim and colleagues identified peptides with angiotensin‐converting enzyme (ACE) inhibitory activity, demonstrating the antihypertensive potential of kefir in a two‐kidney one‐clip model of secondary hypertension that is dependent on the activation of the renin‐angiotensin system (RAS) [42]. The antihypertensive effect of kefir is primarily attributed to the presence of bioactive peptides that exhibit angiotensin‐converting enzyme (ACE) inhibitory properties. This mechanism may contribute to the reduction of blood pressure in individuals consuming kefir [19, 44]. In line with the findings of the present study, probiotic supplementation alone did not lead to significant changes in hs‐CRP levels [45]. Conversely, several earlier studies involving probiotic interventions in different patient populations have reported a decrease in hs‐CRP levels, indicating that the effects may vary based on specific strains, dosages and individual health conditions [46, 47]. The anti‐inflammatory effects of kefir have been assessed in various animal models, showcasing its potential benefits in an animal model of asthma, rats with edema and on the intestinal mucosal immune response in mice [48, 49, 50]. Research involving rats with edema has shown that kefir can effectively reduce inflammation. These findings indicate that kefir may help alleviate symptoms associated with inflammatory conditions, highlighting its potential therapeutic applications [51]. Several studies have found that kefir supplementation can enhance the balance between pro‐inflammatory and anti‐inflammatory cytokines in experimental models of diabetes, metabolic syndrome and obesity [52, 53, 54]. In animal studies, kefir supplementation has been associated with positive changes in metabolic parameters, including lower insulin resistance and reduced fasting glucose levels. These improvements often coincide with beneficial changes in cytokine profiles, indicating a protective effect against inflammation in metabolic syndrome [55]. The positive effects of kefir on cytokine balance may be due to its bioactive components, including peptides and probiotics, which can influence immune responses and help decrease systemic inflammation [56]. The anti‐inflammatory effects of kefir can be explained through both direct and indirect mechanisms that involve the gut microbiota. The indirect mechanisms primarily involve bioactive peptides generated during kefir fermentation, which can stimulate macrophages, enhancing their phagocytic activity and inhibiting the Th2 immune response [57]. Kefir is not only recognised for its probiotic content but also for its rich nutritional profile, which includes various vitamins, minerals, and essential amino acids (EAA). These components may contribute to its anti‐inflammatory properties and other health benefits [58]. The combination of probiotics, B complex vitamins, vitamins C, A, K, carotene, minerals and essential amino acids in kefir may work synergistically to reduce inflammation and promote overall health. Regular consumption of kefir can thus be a beneficial addition to a balanced diet aimed at enhancing well‐being and managing inflammatory conditions. The observed lack of significant changes in health outcomes in some studies related to kefir may be linked to variations in the specific probiotics utilised in the formulations. Different probiotic strains can produce different health effects. For example, kefir that includes specific strains like *Lactobacillus helveticus* and *Bifidobacterium longum* may yield different results compared to standard kefir that lacks these strains [59]. Moreover, the amount of kefir consumed and the length of the study can influence outcomes. Some studies may not have used a sufficient dosage to produce noticeable changes in health markers [56]. Also, the demographics of participants and variation in health condition can lead to inconsistent findings [60].

This systematic review on fermented milk kefir also has several limitations that should be acknowledged when interpreting its findings. The trials included in the review showed a moderate to high risk of bias, which affects the reliability of the reported results and requires careful interpretation. The included trials varied substantially in the age and disease profile of participants; the origin, production method, dosage and duration of kefir interventions; and the control interventions used for comparison. The key concern is the heterogeneity in the control groups used in the included studies. Differences in the types and amounts of supplements given to control groups (e.g., placebo, other dairy products or no supplementation) could have influenced the results. This variability in control group interventions makes it difficult to isolate the specific effect of fermented dairy and hinders the ability to draw definitive conclusions about causality. Due to the significant differences among the trials, conducting a statistical combination of results in a meta‐analysis was deemed inappropriate. However, these findings must be approached with caution due to the small sample sizes associated with these groups, which raise concerns about the potential for type I error. The observed benefits may not be applicable to younger individuals or shorter intervention periods. Due to all these limitations, more long‐term, well‐designed and rigorous RCTs are needed to confirm the role of kefir as a human therapeutic strategy. This study presents several advantages that enhance its value in understanding the effects of fermented milk kefir. It is a thorough systematic review and meta‐analysis that encompasses all RCTs related to kefir, providing a broad overview of the existing evidence. The study did not impose any limitations regarding the publication date or language, which allows for a more inclusive analysis of available research. The use of a standardized methodology is a significant strength of this study. Given the observed heterogeneity among the included studies, the researchers conducted subgroup analyses.

## 5 Conclusion

According to a comprehensive review and meta‐analysis, kefir consumption in adults did not lead to significant reductions in SBP and DBP or CRP levels. However, there is some indication that long‐term consumption of kefir might improve CRP levels over time. These findings suggest that while immediate effects on blood pressure and CRP may not be evident, there could be potential benefits with prolonged use of kefir.

## Author Contributions

Elaheh Rashidbeygi: Data curation, formal analysis, methodology. Mahnoush Mehrzad Samarin: Writing – original draft. Fatemeh Sheikhhossein: Writing – original draft. Masoomeh Gholizadeh: Writing – original draft. Negin Lohrasbi: Writing – original draft. Amin Abbasi: Writing – original draft. Hadi Bazyar: Writing – original draft. Amir Hossein khalilkhaneh: Methodology, writing – original draft. Gholamreza Askari: Writing – review and editing. Mohammad Reza Amini: supervision, writing – review and editing.

## Ethics Statement

The authors have nothing to report.

## Consent

The authors have nothing to report.

## Conflicts of Interest

The authors declare no conflicts of interest.

## Supporting information


**Table S1:** edm270124‐sup‐0001‐TableS1.docx.

## Data Availability

The data that support the findings of this study are available from the corresponding author upon reasonable request.
