# Supplementary material for: The Effect of Kefir Consumption on Blood Pressure and C‐Reactive Protein: A Systematic Review and Meta‐Analysis of Randomised Controlled Trials
Source: Endocrinol Diabetes Metab. 2025 Oct 26;8(6):e70124. doi: 10.1002/edm2.70124 (PMC12554242; doi:10.1002/edm2.70124)
Supplement: Supplementary file 1 — Table S1: edm270124‐sup‐0001‐TableS1.docx. [file EDM2-8-e70124-s001.docx]

**Supplementary Table 1.** Search syntax.

| **Search syntax** | |
| --- | --- |
| (kefir[tiab] OR kefiran[tiab] OR Kefir[Mesh]) AND (hypertension[tiab] OR "Blood Pressure"[tiab] OR Prehypertension[tiab] OR BP[tiab] OR "Systolic blood pressure"[tiab] OR SBP[tiab] OR "Diastolic blood pressure"[tiab] OR DBP[tiab] OR cardiovascular[tiab] OR hypotensive[tiab] OR "Hypertension"[Mesh] OR "Blood Pressure"[Mesh] OR "Prehypertension"[Mesh]) AND (Cytokine[tiab] OR Cytokines[tiab] OR interleukin[tiab] OR interleukins[tiab] OR IL[tiab] OR Inflammation[tiab] OR inflammatory[tiab] OR "C-reactive protein"[tiab] OR CRP[tiab] OR "high‐sensitivity C‐reactive protein"[tiab] OR "high-sensitivity CRP"[tiab] OR hs-CRP[tiab] OR "tumor necrosis factor"[tiab] OR TNF[tiab] OR "tumor necrosis factor-α"[tiab] OR TNF-α[tiab] OR interleukins[tiab] OR Interleukin-1B[tiab] OR IL-1β[tiab] OR "superoxide dismutase"[tiab] OR SOD[tiab] OR Interleukin-6[tiab] OR IL-6[tiab] OR Interleukin-10[tiab] OR IL-10[tiab] OR Interleukin-8[tiab] OR IL-8[tiab] OR Adipokine[tiab] OR Adipokines[tiab] OR "Transforming growth factor beta"[tiab] OR e-selectin[tiab] OR p-selectin[tiab] OR IFN-γ[tiab] OR interferon[tiab] OR "interferon gamma"[tiab] OR Eotaxin[tiab] OR "Inflammation"[Mesh] OR "Cytokines"[Mesh] OR "Interleukins"[Mesh] OR "C-Reactive Protein"[Mesh] OR "Tumor Necrosis Factor-alpha"[Mesh] OR "Adipokines"[Mesh] OR "Interleukin-1beta"[Mesh] OR "Superoxide Dismutase"[Mesh] OR "Interleukin-6"[Mesh] OR "Interleukin-10"[Mesh] OR "Interleukin-8"[Mesh] OR "Transforming Growth Factor beta"[Mesh] OR "E-Selectin"[Mesh] OR "P-Selectin"[Mesh] OR "Interleukin-18"[Mesh] OR "Interferon-gamma"[Mesh])  AND (intervention[tiab] OR RCT[tiab] OR randomized[tiab] OR random[tiab] OR Randomly[tiab] OR Placebo[tiab] OR Assignment[tiab] OR trial[tiab] OR trials[tiab] OR randomised[tiab] OR "Methods"[Mesh] OR Cross-Over[tiab] OR "Double-Blind"[tiab] OR "Randomized Controlled Trial"[Publication Type] OR "Controlled Clinical Trial"[Publication Type] OR "Placebos"[Mesh] OR "Placebo Effect"[Mesh] OR "Clinical Trial"[Publication Type] OR "Clinical Trials as Topic"[Mesh] OR "Cross-Over Studies"[Mesh] OR "Double-Blind Method"[Mesh]) | **Pubmed** |
| ( TITLE-ABS-KEY (kefir) OR TITLE-ABS-KEY (kefiran) AND ( TITLE-ABS-KEY ( hypertension )  OR  TITLE-ABS-KEY ( "Blood Pressure" )  OR  TITLE-ABS-KEY ( prehypertension )  OR  TITLE-ABS-KEY ( bp )  OR  TITLE-ABS-KEY ( "Systolic blood pressure" )  OR  TITLE-ABS-KEY ( sbp )  OR  TITLE-ABS-KEY ( "Diastolic blood pressure" )  OR  TITLE-ABS-KEY ( dbp )  OR  TITLE-ABS-KEY ( cardiovascular )  OR  TITLE-ABS-KEY ( hypotensive ) AND ( TITLE-ABS-KEY ( "interferon gamma" )  OR  TITLE-ABS-KEY ( cytokine )  OR  TITLE-ABS-KEY ( cytokines )  OR  TITLE-ABS-KEY ( interleukin )  OR  TITLE-ABS-KEY ( interleukins )  OR  TITLE-ABS-KEY ( il )  OR  TITLE-ABS-KEY ( inflammation )  OR  TITLE-ABS-KEY ( inflammatory )  OR  TITLE-ABS-KEY ( "C-reactive protein" )  OR  TITLE-ABS-KEY ( crp )  OR  TITLE-ABS-KEY ( "high‐sensitivity C‐reactive protein" )  OR  TITLE-ABS-KEY ( "high-sensitivity CRP" )  OR  TITLE-ABS-KEY ( hs-crp )  OR  TITLE-ABS-KEY ( "tumor necrosis factor" )  OR  TITLE-ABS-KEY ( tnf )  OR  TITLE-ABS-KEY ( "tumor necrosis factor-α" )  OR  TITLE-ABS-KEY ( tnf-α )  OR  TITLE-ABS-KEY ( interleukins )  OR  TITLE-ABS-KEY ( interleukin-1b )  OR  TITLE-ABS-KEY ( il-1β )  OR  TITLE-ABS-KEY ( "superoxide dismutase" )  OR  TITLE-ABS-KEY ( sod )  OR  TITLE-ABS-KEY ( interleukin-6 )  OR  TITLE-ABS-KEY ( il-6 )  OR  TITLE-ABS-KEY ( interleukin-10 )  OR  TITLE-ABS-KEY ( il-10 )  OR  TITLE-ABS-KEY ( interleukin-8 )  OR  TITLE-ABS-KEY ( il-8 )  OR  TITLE-ABS-KEY ( adipokine )  OR  TITLE-ABS-KEY ( adipokines )  OR  TITLE-ABS-KEY ( "Transforming growth factor beta" )  OR  TITLE-ABS-KEY ( e-selectin )  OR  TITLE-ABS-KEY ( p-selectin )  OR  TITLE-ABS-KEY ( ifn-γ )  OR  TITLE-ABS-KEY ( interferon )  OR  TITLE-ABS-KEY ( eotaxin )  AND TITLE-ABS-KEY ( intervention ) OR TITLE-ABS-KEY ( "controlled trial" ) OR TITLE-ABS-KEY ( randomized ) OR TITLE-ABS-KEY ( random ) OR TITLE-ABS-KEY ( randomly ) OR TITLE-ABS-KEY ( placebo ) OR TITLE-ABS-KEY ( assignment ) OR TITLE-ABS-KEY ( "clinical trial" ) OR TITLE-ABS-KEY ( trial ) OR TITLE-ABS-KEY ( randomised ) ) AND ( LIMIT-TO ( DOCTYPE , "ar" ) ) AND ( LIMIT-TO ( LANGUAGE , "English" ) ) AND ( LIMIT-TO ( SRCTYPE , "j" ) ) | **Scopus** |
